# Influence of anesthesia methods on surgical outcomes and renal function in retrograde intrarenal stone surgery: a prospective, randomized controlled study

**DOI:** 10.1186/s12871-019-0901-9

**Published:** 2019-12-23

**Authors:** Ohseong Kwon, Jung-Man Lee, Juhyun Park, Min Chul Cho, Hwancheol Son, Hyeon Jeong, Seung Hoon Ryang, Sung Yong Cho

**Affiliations:** 1 Department of Urology, Hallym University Kangnam Sacred Heart Hospital, Seoul, South Korea; 2 Department of Anesthesiology and Pain Medicine, SMG-SNU Boramae Medical Center, Seoul, South Korea; 3 Department of Urology, SMG-SNU Boramae Medical Center, Seoul, South Korea; 4 Department of Urology, Seoul National University Hospital, 101, Daehak-ro Jongno-gu, 03080 Seoul, Republic of Korea

**Keywords:** Renal stone, Retrograde intrarenal surgery, Spinal anesthesia

## Abstract

**Background:**

We analyzed the influence of anesthesia methods on surgical outcomes and renal function in retrograde intrarenal surgery (RIRS) in a prospective, randomized controlled study.

**Methods:**

Seventy patients who underwent RIRS from September 2015 to February 2017 were randomly allocated to general anesthesia (GA) or spinal anesthesia (SA) groups. Renal function was assessed using estimated glomerular filtration rate, and separate renal function was evaluated using nuclear medicine tests. Maneuverability and accessibility were evaluated after every surgery. All procedures were performed by a single experienced surgeon (SY Cho).

**Results:**

Stone-free rate was higher in the GA (92.3%, 36 of 39) than the SA (71.0%, 22 of 31) (*P* = 0.019) group. Pain score was higher in the GA than in the SA group on the first postoperative morning (*P* = 0.025), but pain scores of the two groups were similar before discharge (*P* = 0.560). There were no differences in the changes of serum creatinine level (*P* = 0.792) and changes of estimated glomerular filtration rate (*P* = 0.807). Differences of separate renal function between operative and contralateral site increased significantly in patients under GA than under SA at postoperative 3 months (*P* = 0.014). Maneuverability and accessibility were better in SA with sedation than GA (*P* < 0.001).

**Conclusions:**

RIRS under SA showed advantages in renal function change using renogram at postoperative 3 months and in lower pain score on the first postoperative morning. Performance of operator under SA was worse than that under GA and significantly improved with sedation. RIRS under SA showed advantages in lower pain score at postoperative first day.

**Trial registration:**

Clinicaltrials.gov ID is NCT03957109, and registration date is 17th May 2019. This study was retrospectively registered.

## Background

According to the European Association of Urology (EAU) guidelines, retrograde intrarenal surgery (RIRS) is recommended as an alternative treatment option to percutaneous nephrolithotomy (PNL) for renal stones less than 2 cm in size [1]. Routinely, most urologists prefer general anesthesia (GA) during surgery for renal stones. Because the patient’s breathing can be controlled, patients can be more comfortable, and the surgeon can be comfortable because of diminished patient movement under general anesthesia. However, some previous studies presented that mortality or major complications are reduced with regional anesthesia than with GA [2–4] and there have been some controversial reports. Although regional anesthesia may be preferred in critically ill patients [5–7], some previous studies, including high quality meta-analyses, suggest that there are no differences in major outcomes or critical complications between GA and spinal anesthesia (SA) [8–10]. Therefore, these two anesthesia methods might be suitable for RIRS in the general population.

Renal function abnormalities or perioperative renal dysfunction due to anesthesia, may be present even in normal patients. A previous study reported that renal dysfunction could happen even in patients who had normal renal function preoperatively [11]. These complications are associated with the type of surgery, baseline renal function, underlying diseases, and the amount of intraoperative bleeding. SA may be free from the toxic effects of muscle relaxants, opioids, and inhalation anesthetics, which may be more beneficial than GA in terms of kidney function [12]. RIRS may increase the risk of electrolyte abnormalities due to absorption of irrigation fluid, and GA presents with limitations in early detection of electrolyte abnormalities. However, there are few previous studies comparing anesthetic methods with respect to renal function [13–15].

Therefore, this study aimed to analyze the influence of anesthesia methods on surgical outcomes and renal function in RIRS.

## Methods

### Patients and study design

**Fig. 1:**
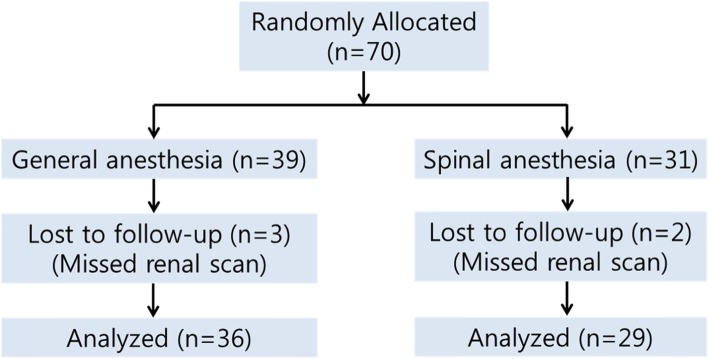
CONSORT diagram

The prospective, randomized study was conducted in compliance with Good Clinical Practices (GCP) and the Declaration of Helsinki, and the study protocol was approved by the Institutional Review Board of Seoul National University Hospital (approval number: 16–2015-75). After receiving written informed consent, 70 patients, classified as American Society of Anesthesiologists (ASA) physical status I-II, registered from September 2015 to February 2017, were recruited. Eligible patients older than 20 years with renal stones greater than 10 mm were included. The decision for active stone removal was based upon the EAU guidelines. Patients were excluded if they had urologic anatomical abnormalities. Patients with ASA status ≥ grade III, a contraindication for spinal anesthesia or RIRS, or unexpected intraoperative renal injury, were also excluded. The CONSORT Statement Extension for Randomized Controlled Trials of Nonpharmacological Trials was closely followed. Following preoperative evaluations, the enrolled patients were randomly allocated to GA or SA groups using the permuted block method. Figure 1 shows the trial design.

### Surgical methods

All RIRS procedures were performed by a single surgeon (SY Cho) who performed over one thousand cases according to the same methods described in the same authors’ investigations [16, 17]. Ureteral access sheaths (Navigator™, Boston Scientific, MA, USA), 11/13-Fr or 12/14-Fr, were inserted at the level of the ureteropelvic junction. Flexible ureteroscopes, Flex-X2S (Karl Storz, Tuttlingen, Germany) and URF-V or V2 (Olympus, Tokyo, Japan), were inserted through the access sheath. After the distal end of the scope was located in the renal pelvis, a 365- or 200-μm laser fiber was used for stone fragmentation. The renal stones were busted into fragments measuring less than 2 mm. An endoscopy irrigation pump (Stryker, Kalamazoo, MI, USA) was used to maintain continuous intrarenal pressure. A double-J stent was routinely inserted at the time of surgery, and removed at postoperative 5 to 10 days.

### Anesthesia methods

Standard monitoring, including non-invasive blood pressure, electrocardiography, oxygen saturation, and body temperature was applied in all patients.

For the GA group, 30 mg lidocaine, 1.5–2.0 mg/kg propofol, 50–100 μg fentanyl was administered to induce anesthesia. Following loss of consciousness, 0.6–0.8 mg/kg rocuronium was administered, and the patients were intubated. Patients were ventilated with a tidal volume of 6–8 ml/kg. Anesthesia was maintained using 1–4 vol% of sevoflurane in oxygen and air.

The patients in the SA group were placed in a lateral decubitus position. An anesthesiologist wearing a sterile gown cleaned the skin on the patient’s back with 0.5% chlorhexidine. After the cleaned skin was dried and draped in a sterile manner, the anesthesiologist inserted a 25-gauge spinal needle into the space between 4th spinous process and 5th spinous process or between 3rd spinous process and 4th spinous process. The intrathecal position of the tip of the needle was confirmed by aspiration of clean cerebrospinal fluid, following which 10–17 mg (median: 14 mg) hyperbaric bupivacaine (Marcaine Heavy inj 0.5%, Astrazeneca, France) and 20 μg fentanyl were administrated intrathecally considering operation site and patient’s height. After injection, the patients were placed in supine position. The anesthesiologist checked for sensory loss in the dermatome, and motor loss to ensure adequate anesthesia for RIRS. On failure of first trial of spinal anesthesia, spinal anesthesia was repeated after patient approval. If the second trial of spinal anesthesia failed, the patient was dropped from the study and general anesthesia was administered in the routine manner. For the SA group, sedation was applied with 1–3 mg of midazolam according to patient’s lead after identifying the level of spinal anesthesia.

### Clinical parameters and statistical analysis

Patient characteristics included age, sex, body mass index, laboratory results, and other comorbidities. Stone characteristics included laterality, Hounsfield units, numbers, maximal size, and volume. Primary outcome was renal function change assessed by estimated glomerular filtration rate and separate renal function evaluated by nuclear medicine test. And, secondary outcome was performance of operator evaluated by one experienced surgeon. The computed tomography (CT) scans were acquired preoperatively. Information regarding the presence of hydronephrosis and infundibulopelvic angles was acquired [18], and the stone distribution was analyzed using the Seoul National University Renal Stone Complexity (S-ReSC) scores [19]. Renal function was assessed using the estimated glomerular filtration rate before surgery and on the day after surgery. Separate renal function was evaluated using nuclear medicine tests with diethylenetriamine pentaacetic acid (99 m Tc-DTPA) prior to procedure, and at 3 months after the procedure. The separate renal function was re-assessed postoperatively, when the difference between the separate renal function of the affected and contralateral sides was over 10%. Intraoperative parameters included operative time and stone fragmentation time per surgery. The operative time was defined as the time from endoscopic insertion into the urethra to the insertion of the urethral Foley’s catheter. Follow-up images of plain X-ray or non-contrast CT were acquired 1, 7, and 60–90 days postoperatively to assess the absence of residual fragments. Stone-free status was clinically defined as the absence of evidence for remnant stones < 2 mm on the follow-up images. Intraoperative incidents associated with anesthesia (blood pressure fluctuation, nausea, and pain) were documented. Blood pressure fluctuation was defined as a change of more than 20% of the baseline systolic blood pressure in the ward before the surgery in this trial.

One experienced surgeon evaluated maneuverability and accessibility after every operation. Maneuverability and accessibility were graded as ‘very poor (1-2)’, ‘poor (3–4)’, ‘so so (5-6)’, ‘good (7–8)’ and ‘very good (9-10)’. Postoperative pain was evaluated using visual analog scale scores from 1 to 10, with 10 being most severe. Postoperative complications were defined according to modified Clavien classification system [20].

We planned a study of experimental subjects with one control(s) per experimental subject. Primary endpoint was difference of renal function recovery rate and the difference was 6.5% from previous investigation. The recovery rate was defined as the rate of changes of separate renal function into less than 10% between the two kidneys in patients who underwent according to the preoperative functional deterioration [11]. In a previous pilot study, the response within each subject group was normally distributed with standard deviation 9%. If the true difference in the previous investigations between the experimental and control means is 6.5%, we will need to study 31 experimental subjects and 31 control subjects to be able to reject the null hypothesis that the population means of the experimental and control groups are equal with probability (power) 0.8 (non-inferiority test). The Type I error probability associated with this test of this null hypothesis is 0.05. Considering dropout rates about 10%, we decided to include total 70 patients (35 patients each).

All parameters were represented as the mean value ± standard deviation or frequency (percentage). Comparative results were analyzed by using an independent *t*-test or a Mann-Whitney U test between the two groups. Categorical variables were analyzed using the Chi-square and Fisher’s exact test. Statistical significance was considered at *P-*value < 0.05. Statistical analyses were performed with IBM SPSS Statistics version 20 (IBM, Chicago, IL, USA) and R version 3.0.1 (http://www.r-project.org).

## Results

### Patients and stone characteristics

Patient demographics and stone characteristics are shown in Table 1. Thirty-nine patients were assigned to the GA group, and 31 patients were assigned to the SA group. All of them completed this study trial. The blockade level of spinal anesthesia was between T2-T10 (number of T2/T3/T4/T6/T8/T10 in the SA group: 2/5/7/8/7/2). One patient complained mild discomfort of pain sensation during the surgery. His height was 178 cm and we injected 14 mg of bupivacaine and 20 μg of fentanyl intrathecally for spinal anesthesia, which lead to a blockade level at T10. We treated the patient with midazolam 5 mg and fentanyl 100 μg because the patient complained of mild discomfort. Baseline preoperative serum creatinine was significantly higher in the GA group than in the SA group (*P* = 0.035). There were no significant differences between the two groups in terms of age, gender, stone laterality, and incidence of comorbidities, and mean number of stones, mean maximal stone size, mean Hounsfield unit, and distribution of S-ReSC scores.

**Table 1:**
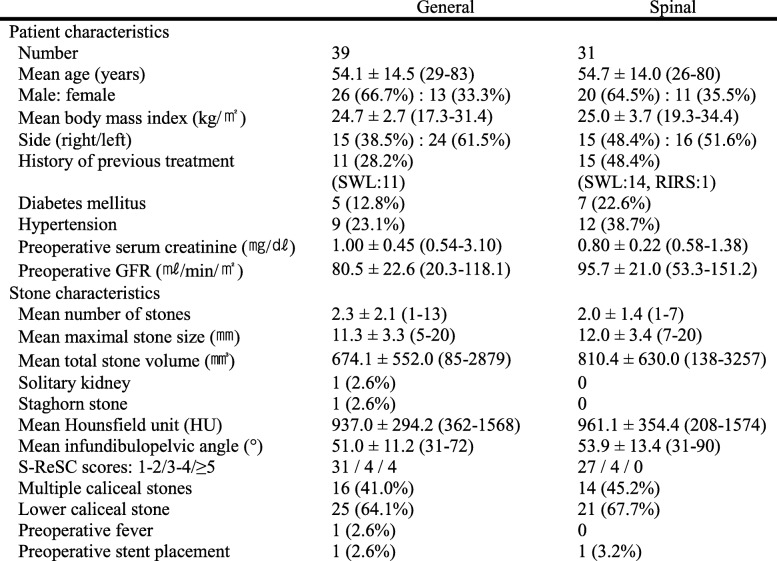
Patient characteristics and perioperative findings

SWL extracorporeal shockwave lithotripsy, RIRS retrograde intrarenal surgery, GFR glomerular filtration rate, S-ReSC Seoul National University Renal stone Complexity

### Surgical outcomes and renal function change

Table 2 shows operative outcomes in the two groups. In the SA group, there were no anesthetic conversions. Stone-free rate (SFR) was higher in the GA (92.3%, 36 of 39) than in the SA (71.0%, 22 of 31) (*P* = 0.019) group. Changes in serum creatinine after surgery were not significantly different between the two groups (*P* = 0.792). Pain score of the GA group was higher than that of the SA group on the first postoperative morning (4.9 ± 2.4 vs. 3.7 ± 1.7, *P* = 0.025); however, pain scores of the two groups were similar before discharge (3.1 ± 1.9 vs. 2.9 ± 1.2, *P* = 0.560) 1 or 2 days postoperatively. There were no differences in postoperative complications between the two groups (*P* = 0.841), and there were no complications of Clavien classification grade III or higher.

**Table 2:**
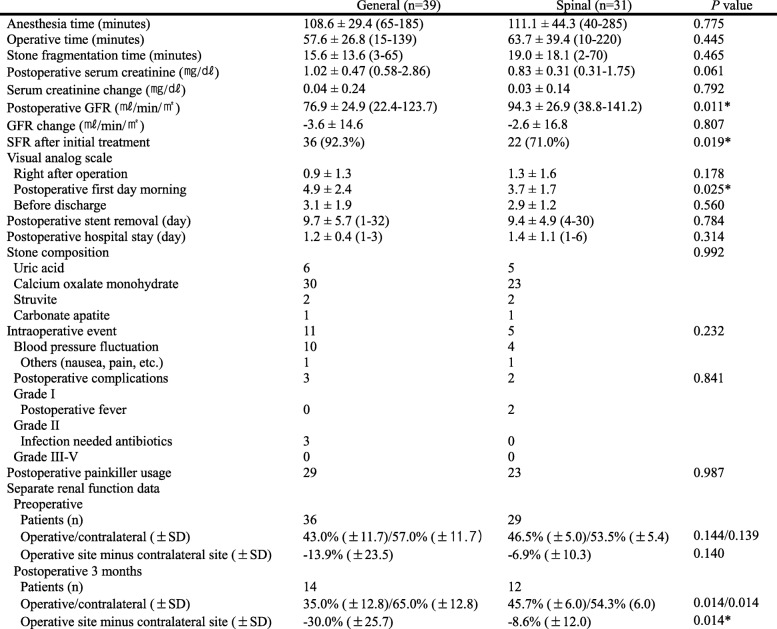
Operative outcomes of the two groups

GFR glomerular filtration rate, SFR stone-free rate, VAS visual analog scale

Considering parameters of renal function after surgery, there were no differences in changes in serum creatinine level (*P* = 0.792) and changes in estimated glomerular filtration rate (*P* = 0.807) between the GA and SA groups. The preoperative DTPA renal scan was performed in 65 of 70 patients, and 26 (40%) showed a difference greater than 10% in separate renal function. After 3 months, 11 of 12 patients (91.7%) in the SA group showed improvement in separate renal function, whereas 10 of 14 patients (71.4%) in the GA group showed improvement in separate renal function (*P* = 0.330). The difference in separate renal function increased significantly in the GA group than in the SA group (*P* = 0.014).

### Maneuverability and accessibility among anesthetic methods

Between GA and SA, scores of maneuverability (5.50 ± 0.00 vs. 5.31 ± 3.44, *P* = 0.726) and accessibility (5.50 ± 0.00 vs. 5.44 ± 3.56, *P* = 0.910) during procedures were not different (Table 3). However, considering sedation during SA, maneuverability and accessibility were better in SA with sedation than in GA, but worse in SA without sedation than in GA. Maneuverability was ‘so so’ (5.50 ± 0.00) in GA, ‘very good’ (9.04 ± 1.20) in SA with sedation, and nearly ‘poor’ (2.61 ± 1.23) in SA without sedation (*P* < 0.001). In addition, accessibility was ‘so so’ (5.50 ± 0.00) in GA, ‘very good’ (9.19 ± 0.75) in SA with sedation, and ‘poor’ (2.72 ± 1.83) in SA without sedation (*P* < 0.001).

**Table 3:** Maneuverability and accessibility among anesthetic methods

	General anesthesia (*n* = 39)	Spinal anesthesia (*n* = 31)	*P* value
Maneuverability			0.726
Very poor (1–2)	0	9	
Poor (3–4)	0	8	
So so (5–6)	39	2	
Good (7–8)	0	1	
Very good (9–10)	0	11	
Accessibility			0.910
Very poor (1–2)	0	11	
Poor (3–4)	0	4	
So so (5–6)	39	2	
Good (7–8)	0	3	
Very good (9–10)	0	11	

*Some SA patients had sedation

## Discussion

The present study compared, for the first time, changes in renal function using renogram after GA with those after SA in RIRS for renal stones. There was no difference in postoperative change in serum creatinine level and estimated glomerular filtration rate between the GA and SA groups. However, patients of SA group showed improvements in separate renal function recovery. The difference in separate renal function between the operative and contralateral sites increased significantly in patients under GA than those under SA 3 months postoperatively. So far other studies concluded that renal function did not worsen postoperatively after they evaluated only serum creatinine level. However, we evaluated separate renal function with DTPA renal scan, and there might be some potential negative effect from GA. Postoperative pain after GA was higher than after SA on the first postoperative morning; however, the pain scores were similar prior to discharge. This study also evaluated maneuverability and accessibility during surgery, and SA with sedation showed better maneuverability and accessibility than GA. In addition, some studies reported that surgeries under regional anesthesia could show reduced major complications (for example, mortality, morbidity, and myocardial infarction) than those under GA [2–4]. In general, urologists routinely prefer GA for RIRS in patients with renal stones. Under GA, surgeons can be more comfortable due to decreased patient movement by controlling breathing [21].

Safety and efficacy of SA compared with that of GA during PNL have been reported several times [22–25]. Karacalar and colleagues suggested that spinal-epidural anesthesia along with intravenous patient-controlled sedation could be an alternative to GA [22]. The incidence of nausea and the use of antiemetics were significantly higher in GA (*P* = 0.01 and 0.001, respectively), and patients reported better satisfaction (*P* = 0.001) and lower pain scores (*P* = 0.001) after spinal-epidural anesthesia. Kuzgunbay and colleagues compared surgical outcomes of PNL between GA and SA [23]. There were no significant differences in SFR, and clinically insignificant residue fragments rates were observed between the two groups (*P* = 0.543). Moreover, there were no significant differences among the surgical parameters (*P* = 0.439); thus, it was suggested that SA did not affect the efficacy and safety of PNL. In 2011, Singh and coworkers performed a prospective and randomized study comparing surgical parameters between GA and SA during PNL [24]. There were no significant differences in complete stone clearance. However, patients after SA used lesser doses of analgesics and had shorter hospital stay. Even in treatment of staghorn stone, PNL was performed safely and effectively under SA [25].

Some studies have reported GA as a risk factor for renal dysfunction after non-urologic surgeries as well [26–28]. An overview of randomized trials commented that regional anesthesia reduced postoperative serious complications [26]. There were reductions in overall mortality, cardiopulmonary complications, and renal failure after SA. Hassan and colleagues investigated renal function change after total hip replacement operation [27]. Patients were classified to have renal impairment using relative increase in serum creatinine level. GA was one of significant risk factors for elevation of serum creatinine levels (*P* = 0.0083). The same authors also reported that GA was a risk factor for renal dysfunction after total knee replacement [28].

Until recently, feasibility of SA for RIRS had been rarely reported. In 2015, Zeng and coworkers conducted a prospective randomized trial to evaluate feasibility of SA for patients undergoing RIRS [13]. There were no significant differences in SFR (*P* = 0.804), postoperative pain (*P* = 0.146), and incidence of complications (*P* = 0.870). Patients in the GA group bore higher medical costs; however, the reports made no comments about sedation during SA. Karabulut and colleagues suggested that SA could be an option for RIRS [13]. There were no significant differences in SFR and postoperative complication rates between SA and GA (*P* > 0.05). Moreover, SA had advantages of lesser postoperative pain and lower medical costs (*P* < 0.001). However, both studies did not evaluate renal function change using renogram after RIRS. In our study, changes in serum creatinine and glomerular filtration rate were not significantly different between the two methods (*P* = 0.792 and 0.807, respectively). The immediate postoperative serum creatinine level did not show the serum creatinine changes due to the short postoperative period. However, the difference of separate renal function between the operative and contralateral sites increased in the GA group at postoperative 3 months (*P* = 0.014). Separate renal function might be affected negatively by GA. Therefore, it might be desirable not to conclude that separate renal function is not deteriorated after only serum creatinine level is evaluated. Present study also assessed maneuverability and accessibility of operator, which were better in SA with sedation than GA. However, the maneuverability and accessibility during SA without sedation were poorer than those during GA, so overall SFR after GA was higher than that after SA in this study.

Although this study had some limitations, to the best of our knowledge, our study was the first prospective, randomized study to compare change in renal function using renogram between SA and GA after RIRS, and to evaluate the surgical performance of the operator. First, the small sample size was a limitation of this study; thus, there was an imbalance between the number of patients after permuted block randomization. Moreover, the authors used the Chronic Kidney Disease Epidemiology Collaboration (CKD-EPI) creatinine-based equation or the Modification of Diet in Renal Disease (MDRD) Study equation in patients with a relatively well-preserved kidney function according to patient status [see Appendix]. The tidal volume during GA was independently chosen by the anesthesiologist, and sedation during SA was decided by patients just before surgery. Further studies comparing GA with low tidal volume and SA with sedation would be helpful to determine a better anesthetic strategy. Maneuverability and accessibility were measured subjectively by the operator, but all measurements were carried out by one experienced operator (SY Cho).

## Conclusions

There were no significant differences in changes in serum creatinine level and estimated glomerular filtration rate between GA and SA groups immediately. Also, operator performance was better under GA than under SA in general. However, RIRS under SA showed advantages in renal function change using renogram at postoperative 3 months and in lower pain score on the first postoperative morning. In addition, the performance of operator was significantly improved under SA with sedation. Hence, this topic warrants further investigations to evaluate safer and more convenient analgesic settings during RIRS.

## Data Availability

The datasets used and/or analyzed during the current study are available from the corresponding author on reasonable request.

## Appendix

Modification of Diet in Renal Disease (MDRD) Study equation.

eGFR(ml/min/1.73 m2) = 186 × (serum creatinine) - 1.154 × (age) - 0.203 × (0.742 if female) × (1.212 if black).

Chronic Kidney Disease Epidemiology Collaboration (CKD-EPI) creatinine-based.

Equation.

eGFR(ml/min/1.73 m2) = 141 × min(Scr/κ,1)^α^ × max(Scr/ κ,1)^-1.209^ × 0.993^Age^ × (1.018 if female) × (1.212 if Black).

κ = 0.7 for females and 0.9 for males.

α = − 0.329 for remales, − 0.411 for males.

min: minimum of Scr/κ or 1.

max: maximum of Scr/κ or 1.

## Abbreviations

CT: computed tomography
EAU: European Association of Urology
GA: general anesthesia
PNL: percutaneous nephrolithotomy
RIRS: retrograde intrarenal surgery
SA: spinal anesthesia
